# Pediatric spinal pilocytic astrocytomas form a distinct epigenetic subclass from pilocytic astrocytomas of other locations and diffuse leptomeningeal glioneuronal tumours

**DOI:** 10.1007/s00401-022-02512-6

**Published:** 2022-10-20

**Authors:** Alice Métais, Yassine Bouchoucha, Thomas Kergrohen, Volodia Dangouloff-Ros, Xavier Maynadier, Yassine Ajlil, Matthieu Carton, Wael Yacoub, Raphael Saffroy, Dominique Figarella-Branger, Emmanuelle Uro-Coste, Annick Sevely, Delphine Larrieu-Ciron, Maxime Faisant, Marie-Christine Machet, Ellen Wahler, Alexandre Roux, Sandro Benichi, Kevin Beccaria, Thomas Blauwblomme, Nathalie Boddaert, Fabrice Chrétien, François Doz, Christelle Dufour, Jacques Grill, Marie Anne Debily, Pascale Varlet, Arnault Tauziède-Espariat

**Affiliations:** 1 Service de Neuropathologie, GHU Psychiatrie et Neurosciences, Site Sainte-Anne, 1 Rue Cabanis, 75014 Paris, France; 2 Institut de Psychiatrie et Neurosciences de Paris (IPNP), UMR_S1266, INSERM, Université de Paris, Equipe IMA-BRAIN (Imaging Biomarkers for Brain Development and Disorders), 102-108 rue de la Santé, 75014 Paris, France; 3 SIREDO Center (Care, Innovation and Research for Children, Adolescents and Young Adults), Institut Curie, Paris, France; 4 Université Paris-Cité, Paris, France; 5 Team Genomics and Oncogenesis of Pediatric Brain Tumors, Molecular Predictors and New Targets in Oncology, INSERM U981, Gustave Roussy, Université Paris-Saclay, Villejuif, France; 6 Pediatric Radiology Department, AP-HP, Hôpital Necker Enfants Malades, Université Paris Cité, Institut Imagine INSERM U1163, 75015 Paris, France; 7 Department of Biostatistics, Institut Curie, PSL University, Paris, France; 8 Department of Biochemistry and Oncogenetic, Paul-Brousse Hospital, Villejuif, France; 9 Aix-Marseille Univ, APHM, CNRS, INP, Inst Neurophysiopathol, CHU Timone, Service d’Anatomie Pathologique et de Neuropathologie, Marseille, France; 10 Département d’anatomie et Cytologie Pathologiques, CHU de Toulouse, IUCT-Oncopole, Toulouse, France; 11 Department of Radiology, Toulouse University Hospital, Toulouse, France; 12 Department of Neurology, Toulouse University Hospital, Toulouse, France; 13 Department of Medical Oncology, IUCT-Oncopole, Toulouse, France; 14 Department of Pathology, CHU Caen, Caen, France; 15 Department of Pathology, CHRU Bretonneau, Tours, France; 16 Department of Neurosurgery, GHU Paris-Psychiatrie et Neurosciences Sainte-Anne Hospital, Paris, France; 17 Department of Pediatric Neurosurgery, Hôpital Necker-Enfants Malades, Assistance Publique Hôpitaux de Paris-Université Paris Cité, Paris, France; 18 Département de Cancérologie de l’Enfant et de l’Adolescent, Institut Gustave Roussy, Université Paris-Sud, Villejuif, France; 19 Univ. Evry, Université Paris-Saclay, Evry, France

**Keywords:** Diffuse leptomeningeal glioneuronal tumour, Pilocytic astrocytoma, Intramedullary glioma, Pediatric low-grade glioma, Glioneuronal tumour, Methylation profiling

## Abstract

Pediatric spinal low-grade glioma (LGG) and glioneuronal tumours are rare, accounting for less 2.8–5.2% of pediatric LGG. New tumour types frequently found in spinal location such as diffuse leptomeningeal glioneuronal tumours (DLGNT) have been added to the World Health Organization (WHO) classification of tumours of the central nervous system since 2016, but their distinction from others gliomas and particularly from pilocytic astrocytoma (PA) are poorly defined. Most large studies on this subject were published before the era of the molecular diagnosis and did not address the differential diagnosis between PAs and DLGNTs in this peculiar location. Our study retrospectively examined a cohort of 28 children with LGGs and glioneuronal intramedullary tumours using detailed radiological, clinico-pathological and molecular analysis. 25% of spinal PAs were reclassified as DLGNTs. PA and DLGNT are nearly indistinguishable in histopathology or neuroradiology. 83% of spinal DLGNTs presented first without leptomeningeal contrast enhancement. Unsupervised t-distributed stochastic neighbor embedding (t-SNE) analysis of DNA methylation profiles showed that spinal PAs formed a unique methylation cluster distinct from reference midline and posterior fossa PAs, whereas spinal DLGNTs clustered with reference DLGNT cohort. *FGFR1* alterations were found in 36% of spinal tumours and were restricted to PAs. Spinal PAs affected significantly younger patients (median age 2 years old) than DLGNTs (median age 8.2 years old). Progression-free survival was similar among the two groups. In this location, histopathology and radiology are of limited interest, but molecular data (methyloma, 1p and FGFR1 status) represent important tools differentiating these two mitogen-activated protein kinase (MAPK) altered tumour types, PA and DLGNT. Thus, these molecular alterations should systematically be explored in this type of tumour in a spinal location.

**Supplementary Information:**

The online version contains supplementary material available at 10.1007/s00401-022-02512-6.

## Introduction

Primary spinal cord tumours represent 2.8–5.2% of the primary central nervous system (CNS) tumours in children and young adults and are most often diagnosed as pilocytic astrocytomas (PA) [29, 30, 36]. It is very likely from the literature that a subset of previously described PA in spinal location might correspond to diffuse leptomeningeal glioneuronal tumours (DLGNT), as up to 62% of published cases of DLGNTs are located in the spine and are often initially misdiagnosed as PA [10, 12, 17, 20, 21, 26, 27, 32, 35, 38, 39, 44, 46]. Distinguishing a PA from a DLGNT represents a diagnostic challenge, particularly in this spinal location, since these two tumours (respectively, circumscribed astrocytic and disseminated glioneuronal) [48], share a similar clinical presentation (a spinal tumuor associated or not with leptomeningeal dissemination at diagnosis) [1, 5, 20, 28, 32, 35], histopathological (oligodendroglial-like morphology with Olig2 and synaptophysin expressions), and molecular features (Mitogen-Associated Protein Kinase (MAPK) pathway alteration) [48]. 1p deletion was previously reported in 59–100% of DLGNTs among this reported series [12, 34]. From a series of 32 DLGNT with DNA-methylation profiling, the 1p deletion was observed in 100% of cases and thus was considered an essential diagnostic criteria in the 2021 WHO classification [48]. However, very little cytogenetic data (only 3 cases reported with 1p19q codeletion) concerning chromosome 1 are available in existing literature (108 reported spinal PAs) (Supplementary Table 1, online resource) [4, 6, 8, 23, 24, 36, 40]. Moreover, there is no series in the literature comparing radiological features and clinical behaviors of these two histomolecular diagnoses in this spinal location. Thus, we performed an integrated radiological, clinico-pathological and molecular (including DNA methylation profiling) characterization of a series of 28 intramedullary LGGs and glioneuronal tumours from patients under the age of 21 years in order to define relevant radiological, pathological and molecular features that would differentiate between PAs from DLGNTs.

## Materials and methods

### Subject selection

We retrieved from the GHU-Paris-Neuro Sainte-Anne database 28 paediatric or adolescents and young adult (AYA) patients operated for a primary spinal tumour with a pathological diagnostic of LGG or glioneuronal tumuor (GNT), non-ependymal, *IDH* and *H3* wildtype, between 2008 and 2020. Written informed consent to participate in this study was provided by the participants’ legal guardian. Data collection was approved under the public health declaration number as folows: DC-2020-3840. Clinical data were retrospectively collected for each case. They included sex, age at diagnosis, tumour location, age at first surgery, extent of surgical resection (gross total resection *vs*. biopsy or partial resection), follow up (including date of first progression radiologically confirmed, treatments, date of last follow up and survival status).

### Radiology data

Initially, post-operative and follow-up magnetic resonance imaging (MRI) were retrieved from Necker Enfants-Malades Hospital database for every patient. Imaging protocols were very heterogeneous as they were realized at different times and in different radiology centers. Spinal studies usually included sagittal SE (Spin Echo) T1-weighted, SE T2-weigthed and SE T1-weighted after gadolinium injection images. Cerebral explorations included T2-weighted, T2-FLAIR (fluid-attenuated inversion recovery), diffusion and T1-weighted with gadolinium injection images. All MRIs were centrally reviewed in consensus by one experienced paediatric neuroradiologist (VDR) and by one radiology resident (WY). Tumour location, extension (height according to the number of adjacent vertebrae), presence of nodular leptomeningeal metastases and leptomeningeal thin enhancement were assessed. Radiological progression was recorded as local recurrence, growth of existing metastases or the appearance of new metastases.

### FISH analyses

Fluorescence in situ hybridization (FISH) studies were performed on interphase nuclei according to the standard procedures and the manufacturer’s instructions and previously published methods [18]. The ploidy for the chromosomes 1 and 19q as well as copy number of the BRAF gene was assessed using the following centromeric and locus-specific probes: Vysis LS1 1p36/1q25 and LS1 19p13/19q13 FISH Probe Kit (Abbott Molecular, USA), ZytoLight^®^ SPEC BRAF Dual Color Break Apart (Zytovision, Germany). NTRK2 rearrangement was assessed using the following centromeric and locus-specific probe: ZytoLight^®^ SPEC NTRK2 Dual color Break Apart (Zytovision, Germany). Signals were scored in at least 100 non-overlapping intact interphase nuclei per case. Gene copy number per nucleus was recorded as follows: one copy, two copies, copy number gain (3–7). Copy gain and deletion were considered if they were detected in more than 10% and 30% of nuclei, respectively. Results were recorded using a DM600 imaging fluorescence microscope (Leica Biosystems, Richmond, IL) fitted with appropriate filters, a CCD camera, and digital imaging software from Leica (Cytovision, v7.4). Normal cells (endothelial cells or normal glial or neuronal cells from the adjacent parenchyma) were used as positive internal controls for locus-specific probes 1p36/1q25 and 19p13/19q13. For BRAF and NTRK2 break apart probes, a positive case with confirmed fusion was used.

### Targeted array SNP genotyping and RNA sequencing data

Molecular data from DNA analysis or RNA sequencing were retrieved from pathological reports. Most experiments were performed according to previously described methods [31, 42]. Briefly, total DNA was extracted with the use of the QIAampDNA mini-kit^®^ (Qiagen Inc., Courtaboeuf, France) according to manufacturer’s protocols. Briefly, tissues were disrupted in lysis buffer. After removing paraffin, the DNA was purified via sequential centrifugation through membrane spin columns. The purity and quantity of DNA were assessed by measuring the absorbance ratio at 260/280 nm with a NanoDrop^®^ Spectrophotometer (LabTech, Palaiseau, France). A brain tumour gene mutation panel was developed using the MassARRAY iPlex technology and MassARRAY online design tools (Agena Bioscience), including the following mutations: *IDH1* R132HLSGC; *IDH2* R172KTMGWS; *BRAF* V600EGAKRD; *EGFR* A289TSVD, D770ins; *FGFR1* K656EQ, N546K; *H3F3A* K27M, G34VRW; *HIST1H3B* K27MT; *HISTH3C* K27M, *TERT* promoter c228at, c250t. The MassARRAY iPlex procedure involves a three-step process consisting of the initial polymerase chain reaction (PCR), inactivation of unincorporated nucleotides by shrimp alkaline phosphatase and a single-base primer extension. Then, the products are nano-dispensed onto a matrix-loaded silicon chip (SpectroChipII, Agena Bioscience, San Diego, California, USA). Finally, the mutations are detected by MALDI–TOF (matrix-assisted laser desorption-ionization–time of flight) mass spectrometry. Data analysis was performed using MassARRAY Typer Analyzer software 4.0.4.20 (Agena Bioscience, San Diego, California, USA), which facilitates visualization of data patterns as well as the raw spectra.

RNA was extracted from two 8-μm-thick formol-fixed paraffin-embedded (FFPE) material sections using the high Pure FFPE RNA Isolation Kit (Roche Diagnostics, Boulogne-Billancourt, France) according to the manufacturer’s instruction. RNA concentrations were measured on a Qubit 4 Fluorometer (Thermo Fisher Scientific, USA) with the Invitrogen Qubit RNA BR Kit (Thermo Fisher Scientific). The percentage of RNA fragments > 200 nt (fragment distribution value; DV200) was evaluated by capillary electrophoresis (Agilent 2100 Bioanalyzer). A DV200 > 30% was required to process the next steps in the analysis. Next-generation sequencing (NGS)-based RNA sequencing was performed using the Illumina TruSight RNA Fusion Panel on a NextSeq550 instrument according to the manufacturer’s instructions (Illumina, San Diego, CA, USA). This targeted RNA sequencing panel covers 507 fusion-associated genes, to assess the most known cancer-related fusions. The TruSight RNA Fusion Panel gene list is available at https://www.illumina.com/content/dam/illumina-marketing/documents/products/gene_lists/gene_list_trusight_rna_fusion_panel.xlsx. A total of 7690 exonic regions are targeted with 21,283 probes. Libraries were prepared according to the Illumina instructions for the TruSight RNA Fusion Panel kit. STAR_v2.6.1a or Bowtie software was used to produce aligned reads in relation to the Homo sapiens reference genome (UCSC hg19) [13]. Manta v1.4.0, TopHat2 and Arriba tools were used for fusion calling [9].

### Droplet digital PCR

*FGFR1* tyrosine kinase domain (TKD) duplication and hotspot mutations (N546K/K656E) were assessed by previously described droplet digital polymerase chain reaction (ddPCR) [2, 15, 16]. Extracted DNA was quantified using the IDQUANTq kit (ID-Solutions, Grabels, France) with the magnetic induction cycler (Mic) PCR Machine Cycler from Bio Molecular Systems (Göttingen, Germany). After quantification, DNA concentration was adjusted. Eight microliters of DNA comprising 1–5 ng and 14 µl of PCR mix (ready to use) were used for each ddPCR assay. A similar amplification program (50 °C 2 min; 95 °C 10 min; 40 × 95 °C 30 ss–60 °C 1 min; 98 °C 10 min) was used for all targets. The QX200 Droplet Digital PCR System (Bio-Rad, Hercules, California, USA) was used with the AutoDG droplet generator (Bio-Rad). Quantasoft Analysis Pro Software v1.0.596 (Bio-Rad) was used for the qualitative and quantitative analyses. Fractional abundance and copy number variations (CNV) were calculated with the cut-off values and detection thresholds defined by Appay et al. [2]. The cut-off value of positive results for mutant detection were two positive droplets. Detection thresholds were set when the number of positives droplets was strictly above the limit of blank at 95% confidence interval defined for each assay depending on the number of replicates.

### DNA methylation profiling data

Nineteen tissue samples (FFPE or freshly frozen if available), for which 500 ng of DNA was extracted were analyzed. DNA was extracted using the QIAamp^®^ DNA Tissue kit or QIAamp^®^ DNA FFPE Tissue Kit (Qiagen, Hilden, Germany) for FFPE samples. DNA from FFPE samples was restored using the Infinium HD FFPE Restore Kit (Illumina, San Diego, California, USA). Bisulfite conversion was performed using the Zymo EZ DNA methylation Kit (Zymo Research, Irvine, California, USA). Standard quality controls confirmed DNA quality/quantity and bisulfite conversion. DNA was then processed using the either Illumina Infinium Methylation EPIC or HumanMethylation450 BeadChip (Illumina, San Diego, California, USA) arrays according to the manufacturer’s instructions. The iScan control software was used to generate raw data files in.idat format, analyzed using GenomeStudio software version v2011 and checked for quality measures according to the manufacturer’s instructions.

Affiliation predictions to CNS tumour classes were obtained from a DNA methylation-based classification of CNS tumours from DKFZ (Deutsches Krebsforschungszentrum—German Cancer Research Center) based on a random forest algorithm available on the web platform www.molecularneuropathology.org. Version v12.5 of the algorithm was used for the present study. The output of this classifier is a score (calibrated score, CS) indicating the resemblance to the reference CNS tumour class in the algorithm. We choose a dimension reduction technique for data visualization: the t-SNE algorithm (t-distributed stochastic neighbor embedding). This non-linear method allows the visualization of data in the form of scatter plots and is well suited for the analysis of raw methylation data. Distinct samples from the same tumour type will usually lead to compact clusters. However, there is no distance threshold that can serve to determine if one sample of interest belongs to one particular cluster; we thus consider that a sample belongs to one class of reference if it overlaps the corresponding cluster or fell in the close vicinity. This method is frequently used in cancer research and to study DNA methylation profiling data in CNS tumours. It was used in the original paper on the classification of central nervous system tumours based on DNA methylation profiles by Capper et al. [7]. Parameters used in this study are the same as those from the DKFZ.Data from EPIC and 450k methylation array were analysed with R language (v4.0.4). The minfi package was used to load idat file and preprocessed with function preprocess.illumina with dye bias correction and background correction. We removed probes located on sex chromosomes, not uniquely mapped to the human reference genome (hg19), probes containing single nucleotide polymorphisms and probes that are not present in both EPIC and 450k methylation array. A batch effect correction was done with removebatchEffect function from limma package, to remove difference between FFPE and frozen samples. The probes were sorted by standard deviation with 10,000 most variable probes were kept for the clustering analysis. These probes were used to calculate the 1-variance weighted Pearson correlation between samples. The distance matrix was used as input for t-distributed stochastic neighbor embedding (t-SNE) from Rtsne package, with the following non-default parameters: theta = 0, pca = F, max_iter = 2500 and perplexity = 20. Visualization was done using ggplot2 packages.

### CNV analysis of KIAA1549:BRAF fusion and 1p deletion

*KIAA1549:BRAF* and 1p deletion were searched by visual inspection of CNV profiles generated by the molecularneuropathology.org platform as described in Stichel et al. [43]. Visual inspection indicated a deletion of 1p if a complete loss of chromosome 1p arm was present. A gain of 7q34 region was indicative of the *BRAF* duplication and *KIAA1549:BRAF* fusion.

### Histopathological analysis, immunohistochemistry and integrated diagnostic

Samples were stained with hematoxylin-phloxin-saffron (HPS) according to standard protocols. Original slides from all tissue samples were centrally reviewed (ATE, AM) on a Nikon Eclipse E600 (Nikon, Japan) light microscope with Nikon Plan Fluor objectives. Due to often limited examinable surface, mitotic activity was monitored on five high-power fields (HPF, 40 ×/0.75) corresponding to 1.6 mm^2^. The following primary antibodies were used: Glial Fibrillary Acidic Protein (GFAP) (1:200, clone 6F2, Dako, Glostrup, Denmark), Olig2 (1:3000, clone C-17, Santa Cruz Biotechnology, Dallas, USA), CD34 (1:40, clone QBEnd10, Dako, Glostrup, Denmark), Chromogranin A (1:200, clone LK2 H10, Diagnostic Biosystem, Pleasanton, USA), Neurofilament Protein (1:100, clone 2F 11, Dako, Glostrup, Denmark), Synaptophysin (1:150, clone DAK-SYNAP, Dako, Glostrup, Denmark), Ki-67 (1:200, clone MIB-1, Dako, Glostrup, Denmark). Integrated diagnoses were performed according to the current WHO classification [48].

### Statistics

Quantitative variables are expressed by median and compared using Mann–Whitney tests. Qualitative variables are expressed by proportions and percentages and compared using Fischer’s exact test. Those analyses were performed in GraphPad Prism version 7.0a. The Reverse Kaplan–Meier method was used to determine the median follow up [37]. Time to treatment initiation (TTI) was calculated as the duration of time between diagnosis and the initiation of first treatment. Progression-free survival, termed PFS-R, was defined as the time between first treatment start and first radiologically confirmed progression on magnetic resonance imaging (MRI). Overall survival (OS) was calculated as the time from the date of diagnosis to the date of death from any cause or the date of last follow-up. Patient were considered disease-free (DF) if gross total resection was achieved and without any signs of disease recurrence at last follow-up. The appearance of new leptomeningeal contrast enhancement and/or of distant metastases, and/or the growth of the main tumour site, as assessed by MRI, were considered as disease progression (DP). Censored variables were analyzed using the Kaplan–Meier method and comparison were assessed using the log-rank test performed in R version 4.1.2 [33]. All statistical significance was considered at a 5% alpha level. Values between brackets [] are 95% confidence intervals unless stated otherwise.

## Results

### 25% of spinal PAs were reclassified as DLGNTs

**Fig. 1:**
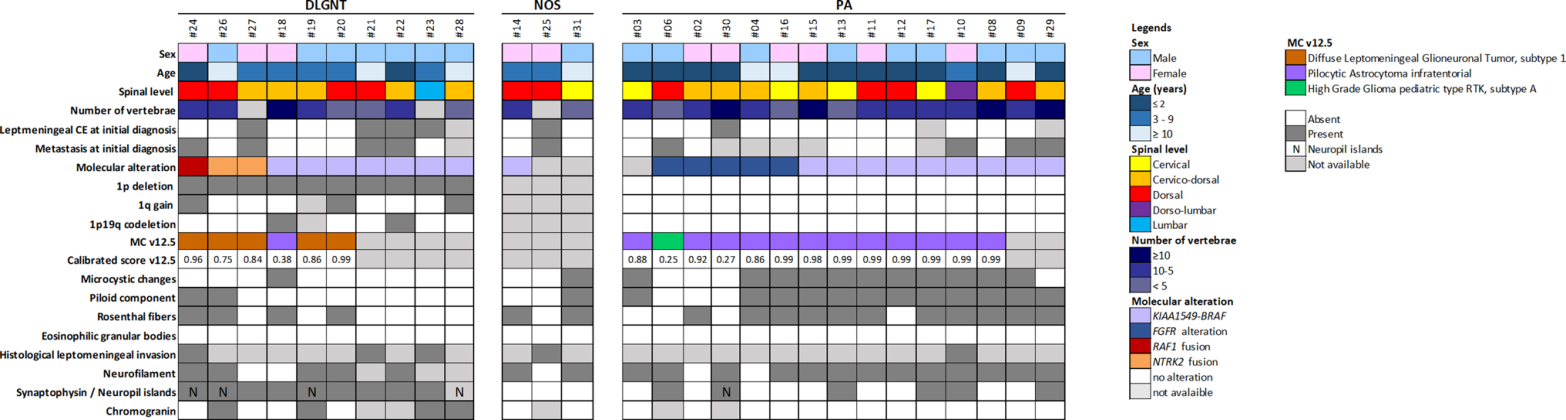
Characteristics of primary pediatric primary spinal low grade glioma and glioneuronal tumours. PA diagnosis was made for cases presenting as, either a low-grade piloid astrocytic neoplasm with solitary MAPK alteration and without 1p deletion (13/15 cases), or with a DNA methylation profile clustering with PA on t-SNE analysis despite a low calibrated score (< 0.9) for PA methylation class” and DLGNT diagnosis was made for oligodendroglioma-like tumours with Olig2 and neuronal immunoreactivity and a MAPK alteration associated with chromosome arm 1p deletion. A diagnosis of LGG NOS was made when molecular analysis (particularly 1p status and DNA methylation profiling) could not be fully performed or failed technically due to low DNA quality and/or quality. Copy number alterations were assessed by FISH assays and corroborated by methylation data. Molecular alterations were detected by FISH assays for *BRAF* rearrangement, *FGFR1* alterations were assessed by ddPCR or NGS, *RAF1* and *NTRK2* fusions were assessed by RNA sequencing. DLGNT subtype 1 refers to a methylation class according to Deng et al. assigned by the version 12.5 of DKFZ classifier. High Grade glioma pediatric type RTK is also a methylation class assigned by the classifier that is further split up into 3 subtypes A, B and C that are currently not fully understood. The methylation class infratentorial pilocytic astrocytoma represents pilocytic astrocytoma that are located in the posterior fossa and that carry MAPK pathway alterations. *CE* contrast enhancement, *DLGNT* diffuse leptomeningeal glioneuronal tumour, *LGG* low-grade glioma, *MAPK* mitogen-activated protein kinase, *MC v12.5* methylation class according to the version 12.5 of the *molecularneuropathology.org* CNS tumour classifier, *N* neuropile islands, *NOS* not otherwise specified, *PA* pilocytic astrocytoma

Integrated diagnoses took into account WHO criteria and DNA methylation profiling analysis which includes t-SNE analysis [7, 48]. PA diagnosis was made for cases presenting as either a low-grade piloid astrocytic neoplasm with solitary MAPK alteration and without 1p deletion (13/15 cases), or with a DNA methylation profile clustering with PA on t-SNE analysis despite a low calibrated score (as defined as < 0.9 in Capper et al. [7]) for PA methylation class (MC) (2 infantile cases with FGFR1 without 1p deletion). DLGNT diagnosis was made for oligodendroglioma-like tumours with Olig2 and neuronal immunoreactivity and a MAPK alteration associated with chromosome arm 1p deletion. Spinal tumours were reclassified as follows: (1) DLGNTs accounted for 36% (10/28) of the cohort (initially diagnosed by original pathologists as 7 PA, one ganglioglioma, one oligodendroglioma not otherwise specified (NOS), and one glioneuronal tumour NOS). PAs accounted for 54% (15/28) (initially diagnosed by original pathologists as 13 PAs, one pilomyxoid astrocytoma, one oligodendroglioma). Three cases remained unclassified and, therefore, were diagnosed as spinal pediatric LGG NOS because molecular analysis could not be fully performed or failed technically due to low DNA quality and/or quality (Fig. 1 and Supplementary Table 2, online resource).

### MAPK alterations in spinal DLGNTs do not include FGFR1 mutations or internal tandem duplication

**Fig. 2:**
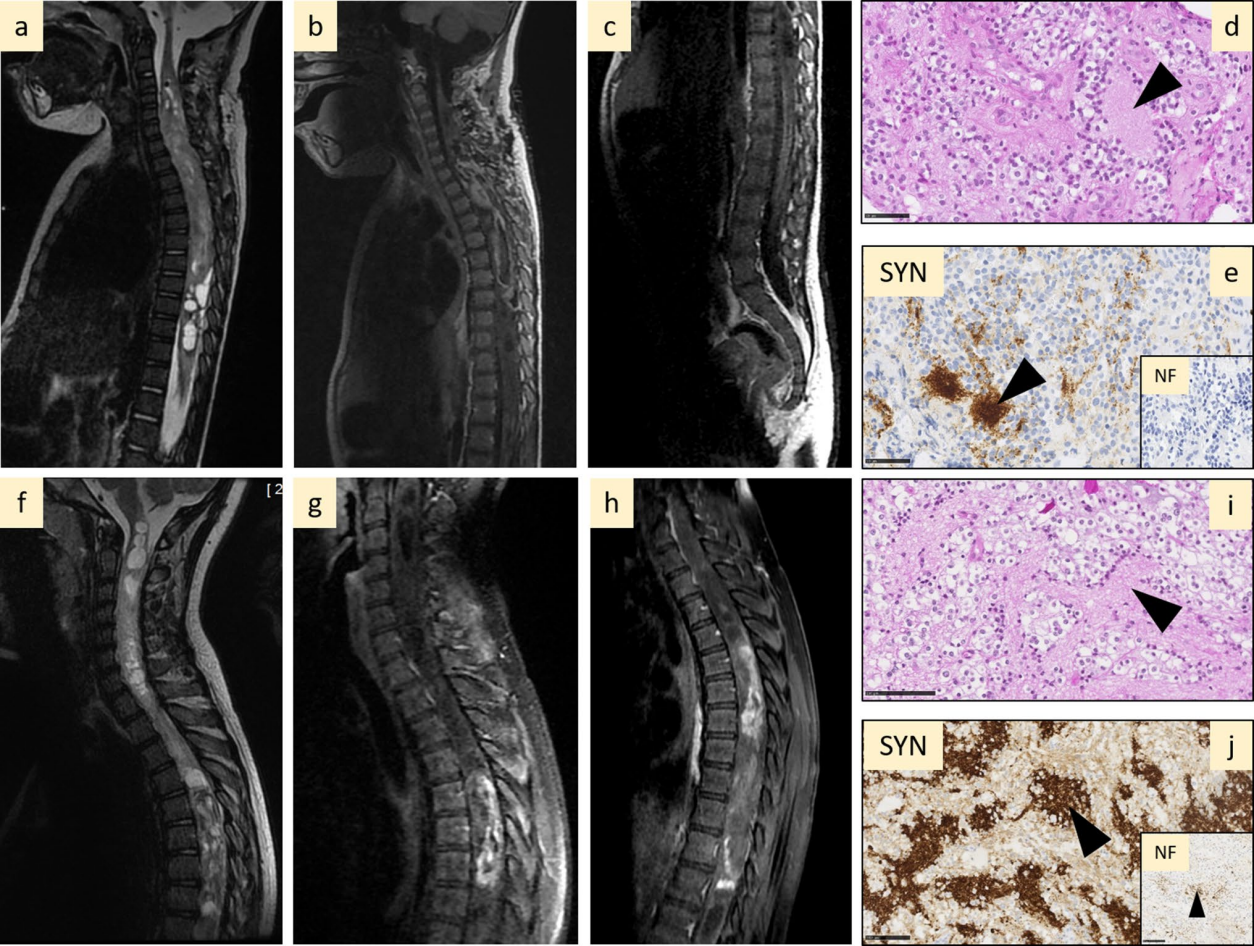
Radiological and histopathological aspects of pilocytic astrocytoma and diffuse leptomeningeal glioneuronal tumour. **a**–**e** Illustration of case #30, a PA *FGFR1* altered with leptomeningeal contrast enhancement at initial diagnosis and neuropil islands: sagittal T2-weighted (**a**) and T1-weighted after contrast injection (**b**, **c**) images showing a heterogeneous cystic and nodular tumour with partial contrast enhancement, and leptomeningeal enhancement along the cauda equina nerve roots, synaptophysin positive neuropil islands (arrow head, **d**, **e**) in neurofilament negative areas (zoom box **e**). **f**–**j** Illustration of case #26, a DLGNT with *NTRK2:AGAP1* fusion and without leptomeningeal contrast enhancement: sagittal T2-weighted (**f**) and T1-weighted after contrast injection (**g**, **h**) images showing a heterogeneous cystic and nodular tumour with partial contrast enhancement, without leptomeningeal involvement, synaptophysin positive neuropil islands (arrow head **i**, **j**). **d**, **e** Scale bar = 50 µm; **i**, **j**: scale bar = 100 µm. *DLGNT* diffuse leptomeningeal glioneuronal tumour, *PA* pilocytic astrocytoma

Whereas PAs and DLGNTs share a frequent *KIAA1549:BRAF* fusion (64% (*n* = 9/14) in PAs and 70% (*n* = 7/10) in DLGNTs), *FGFR1* alterations (3 TKD mutations and 2 internal tandem duplication, ITD) were exclusively observed in PA [33% (*n* = 5/15)] (Fig. 1, case #30 illustrated in Fig. 2a–e). Among DLGNT, 30% (*n* = 3/10) had rare fusion transcripts: one *QKI:RAF1* fusion and two *NTRK2* fusion (*NTRK2:AGAP1* and *TNS3:NTRK2*) (Fig. 1, case #26 illustrated in Fig. 2f–j). 1q gain was observed in 3 cases (Fig. 1 and Supplementary Table 2, online resource). There was a complete correspondence between the visual analysis of the CNV plots obtained from the DKFZ (Deutsches Krebsforschungszentrum—German Cancer Research Center) classifier and the FISH results, both for the chromosome 1 CNVs and BRAF rearrangement (examples are provided in Supplementary Fig. 1, online resource).

### Spinal PAs are epigenetically distinct from others PAs and DLGNTs

**Fig. 3:**
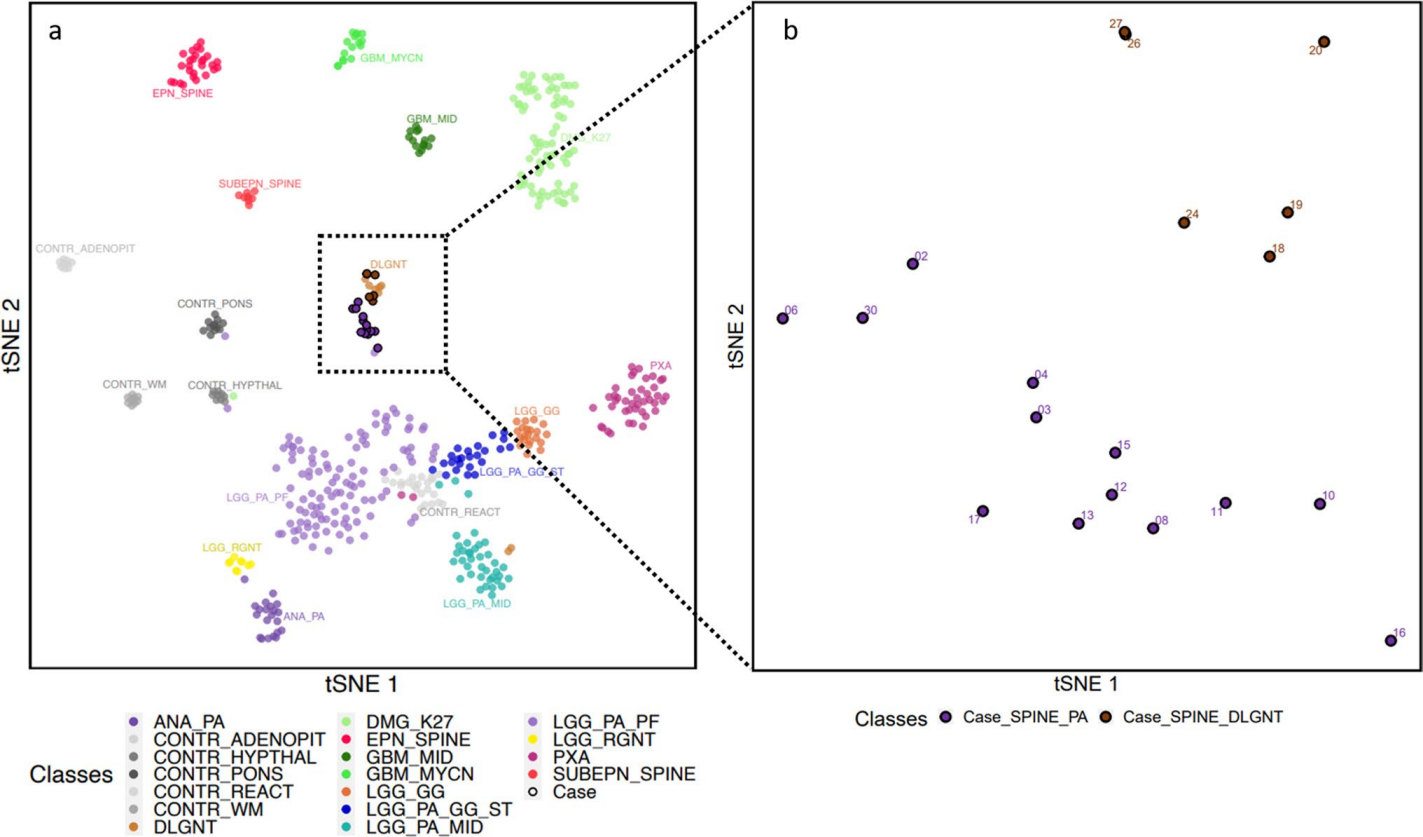
t-SNE clustering analysis of pediatric primary spinal low grade glioma and glioneuronal tumours against the DKFZ reference dataset. The 13 spinal PAs cases analyzed by DNA methylation profiling formed a unique cluster distinct from hemispheric pilocytic astrocytomas and gangliogliomas (LGG_PA_GG_ST), posterior fossa pilocytic astrocytomas (LGG_PA_PF) and midline pilocytic astrocytomas (LGG_PA_MID) as well as from DLGNTs (**a**, **b**). Cases: *n* = 19. ANA_PA: methylation class anaplastic pilocytic astrocytoma (*n* = 21); CONTR_ADENOPIT: methylation class control tissue, pituitary gland anterior lobe (*n* = 9); CONTR_HYPTHAL: methylation class control tissue, hypothalamus (*n* = 9); CONTR_PONS: methylation class control tissue, pons (*n* = 12); CONTR_REACT: methylation class control tissue, reactive tumour microenvironment (*n* = 23); CONTR_WM: methylation class control tissue, white matter (*n* = 9); DLGNT: methylation class diffuse leptomeningeal glioneuronal tumour (*n* = 8; 5/8 reference cohort samples had a spinal location, they were centrally reviewed in Heidelberg and initial diagnoses were diffuse leptomeningeal glioneuronal tumour, age at diagnosis ranged from 5 to 39 years old, including 2 adults of 26 and 39 years old); DMG_K27: methylation class diffuse midline glioma H3 K27M mutant (*n* = 78); EPN_SPINE: methylation class ependymoma, spinal (*n* = 27); GBM_MID: methylation class glioblastoma, IDH wildtype, subclass midline (*n* = 14); GBM_MYCN: methylation class glioblastoma, IDH wildtype, subclass MYCN (*n* = 16); LGG_GG: methylation class low grade glioma, ganglioglioma (*n* = 21); LGG_PA_GG_ST: methylation class low grade glioma, subclass hemispheric pilocytic astrocytoma and ganglioglioma (*n* = 24); LGG_PA_MID: methylation class low grade glioma, subclass midline pilocytic astrocytoma (*n* = 38); LGG_PA_PF: methylation class low grade glioma, subclass posterior fossa pilocytic astrocytoma (*n* = 114); LGG_RGNT: methylation class low grade glioma, rosette forming glioneuronal tumour (*n* = 9); PXA: methylation class (anaplastic) pleomorphic xanthoastrocytoma (*n* = 44); SUBEPN_SPINE: methylation class methylation class subependymoma, spinal (*n* = 9).

A t-SNE dimensionality reduction analysis was performed on the DNA methylation profiles of 13 spinal PAs and 6 spinal DLGNTs, in comparison with cases from the DFKZ reference cohort (Fig. 3a, b). The 13 spinal PAs cases analyzed by DNA methylation profiling formed a unique cluster distinct from hemispheric pilocytic astrocytomas and gangliogliomas (LGG_PA_GG_ST), posterior fossa pilocytic astrocytomas (LGG_PA_PF) and midline pilocytic astrocytomas (LGG_PA_MID) as well as from DLGNTs. The 6 DLGNT cases clustered together with the DKFZ DLGNT reference cohort, whatever their location and their chromosome arm 1q status. Methylation classes and calibrated scores are detailed in Fig. 1 and Supplementary Table 2, online resource. Of note, one case (#18) with *KIAA1549:BRAF* and a 1p deletion had a MC of PA_INF with a calibrated score of 0.38 but the t-SNE nonetheless reclassified it as a DLGNT (Figs. 1, 2). Another case (#06), with a FGFR1 alteration, without 1p deletion, and a MC of diffuse pediatric high-grade glioma, RTK1 type, subtype A with a calibrated score of 0.25 was reclassified as a spinal PA by t-SNE analysis (Figs. 1, 3 and Supplementary Fig. 3, online resource).

### Histopathology alone do not distinguish PAs and DLGNTs in spinal locations

Microcystic changes and piloid component were significantly more frequently observed in PAs (respectively, *p* = 0.003 and *p* = 0.005) (Fig. 1, Table 1 and Supplementary Fig. 3, online resource). Neuropil islands were observed in 40% (*n* = 4/10) of DLGNT (Fig. 1, Table 1, Fig. 2i, j) and only one in PA (7%, *p* = 0.120) (Table 1, Fig. 2d, e). Rosenthal fibers were present in 73% of PA and 40% of DLGNT; however, no statistical difference was observed (*p* = 0.122) (Table 1 and Supplementary Fig. 3, online resource). No case displayed eosinophilic granular bodies. The expression of neuronal markers (synaptophysin and chromogranin A) was significantly more often observed in DLGNTs than PAs (respectively, *p* = 0.009 and *p* = 0.011) (Fig. 1, Table 1). There was no noticeable difference in terms of vasculature between the two tumour types (Table 1). Glial markers, Olig2 and GFAP, were expressed in both tumour types (Table1). No statistical difference of mitotic count or Ki67 index was demonstrated between PAs and DLGNTs (Table 1). Median mitotic count was 1 [0; 9] mitoses per 1.6 mm^2^ for PAs and 2.5 [0; 7] mitoses per 1.6 mm^2^ for DLGNTs. The median Ki67 index of proliferation was 4% [3; 15] in PAs and it was 6% [1; 10] in DLGNTs (Table 1).

**Table 1:** Clinico-radiological and histopathological data of PA and DLGNT

	PA	DLGNT	*p*	Significance
Age (median, years)	2	8.5	**0.010**	*
Progression	47%	77%	0.209	ns
Number of vertebrae (median)	7	6	0.693	ns
Leptomeningeal contrast enhancement	7%	44%	0.260	ns
Metastasis at initial diagnosis	26%	44%	0.371	ns
Microcystic changes	73%	10%	**0.003**	**
Piloid component	80%	20%	**0.005**	*
Neuropil islands	7%	40%	0.120	ns
Rosenthal fibers	73%	40%	0.122	ns
Hyalin vessels	53%	50%	> 0.999	ns
Microvascular proliferation	73%	50%	0.397	ns
Barrel-shaped vessels	27%	20%	> 0.999	ns
Mitotic count (median)	1	2.5	0.521	ns
GFAP	93%	62%	0.272	ns
Synaptophysin	47%	100%	**0.009**	**
Chromogranin A	0%	50%	**0.011**	*
Ki67	4%	6%	0.981	ns

Significant values are in bold

### Radiological characteristics of spinal PAs and DLGNTs

MRI did not allow distinction between PA and DLGNT, regarding primary tumour characteristics as well as leptomeningeal dissemination (Table 1). Both tumour types were intramedullary cystic and solid with high T2-weighted signal intensity and heterogeneous contrast enhancement (CE). At initial diagnosis, the intramedullary tumour extension was similar in both tumour types and was most often located in the cervico-thoracic region (median number vertebrae involved was 7 in PAs and 6 in DLGNTs, *p* = 0.693, Fig. 1, Table 1, and Supplementary Table 2, online resource). Metastatic dissemination at the time of initial diagnosis was observed in 26% (*n* = 4/15) PAs and 44% (*n* = 4/9) DLGNTs (Fig. 1). At the initial diagnosis, a thin leptomeningeal CE was observed in 1/13 PAs (8%) (case #30, Fig. 3a–c), and in 4/9 DLGNTs (44%). Five DLGNTs did not have a leptomeningeal CE at initial diagnosis (56%) (case #26, Fig. 3f–h). PA and DLGNT demonstrated no statistical difference in terms of metastasis and leptomeningeal CE. Radiological progression was a frequent event in both groups, and was most commonly accounted for a local progression of the disease (Table 1, Supplementary Table 2, online resource). Patients who were initially diagnosed as non-metastatic did not show leptomeningeal dissemination during follow-up (median follow-up was 61 [2.4; 134.2]). Progression of existing metastasis or occurrence of new metastases occurred respectively in 20% (*n* = 2/10) and 10% (*n* = 1/10) of DLGNTs (Table 1, Supplementary Table 2, online resource).

### Demography and survival analyses

**Fig. 4:**
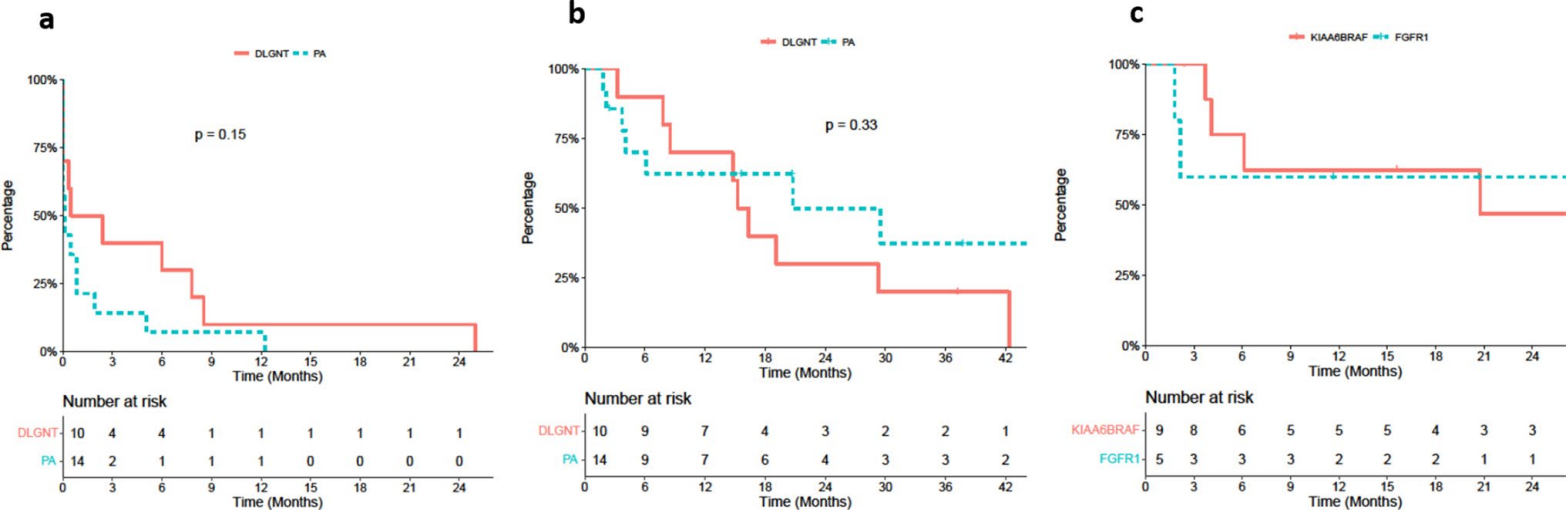
Survival analyses. No statistical differences could be evidenced between PA and DLGNT for the time to treatment (TTI, **a**) and the Progression-Free Survival (PFS-R, **b**) nor between the *KIAA1549:BRAF* altered PA versus *FGFR1* altered PA (PA PFS-R, **c**)

Sex ratio in the entire cohort of 28 cases was 1.5 (male: female), with no significant differences between DLGNTs and PAs. Overall median age at diagnosis was 4 years [1; 18]. 46% (*n* = 13/28) of the cases were infants (≤ 3 years), 46% (*n* = 13/28) were children (4–17 years) and 8% (*n* = 2/28) were AYA (18–25 years). PAs occurred in significantly younger children than DLGNTs: median age at diagnosis was 2 [range 0.7; 13] years for PA and 8.5 [range 1.9; 18] years for DLGNTs (*p* = 0.016) (Table 1 and Supplementary Table 2, online resource). Overall median follow-up was 61 [range 2–134] months. Two patients died, one from disease progression 14 months after diagnosis (a NOS case) and the second from Sars-Cov2 infection 3 years and 4 months after diagnosis (a DLGNT case). First-line treatment modalities comprised surgery, chemotherapy or both, and were partitioned similarly between the DLGNT and the PA groups (Supplementary Table 3, online resource). The mean TTI was equal for both groups (Fig. 4a). Since the therapeutic approach was similar in both groups, the survival rates were easily compared. The overall median PFS-R was 19 months [9; NA], without significant differences between the PA and DLGNT groups (respectively, 21[4; NA] and 16[9; NA] months) (Fig. 4b). Within the PA group, the 5 *FGFR1* altered cases and the 9 *KIAA1449:BRAF* rearranged cases showed no differences in terms of survival (Fig. 4c).

## Discussion

**Fig. 5:**
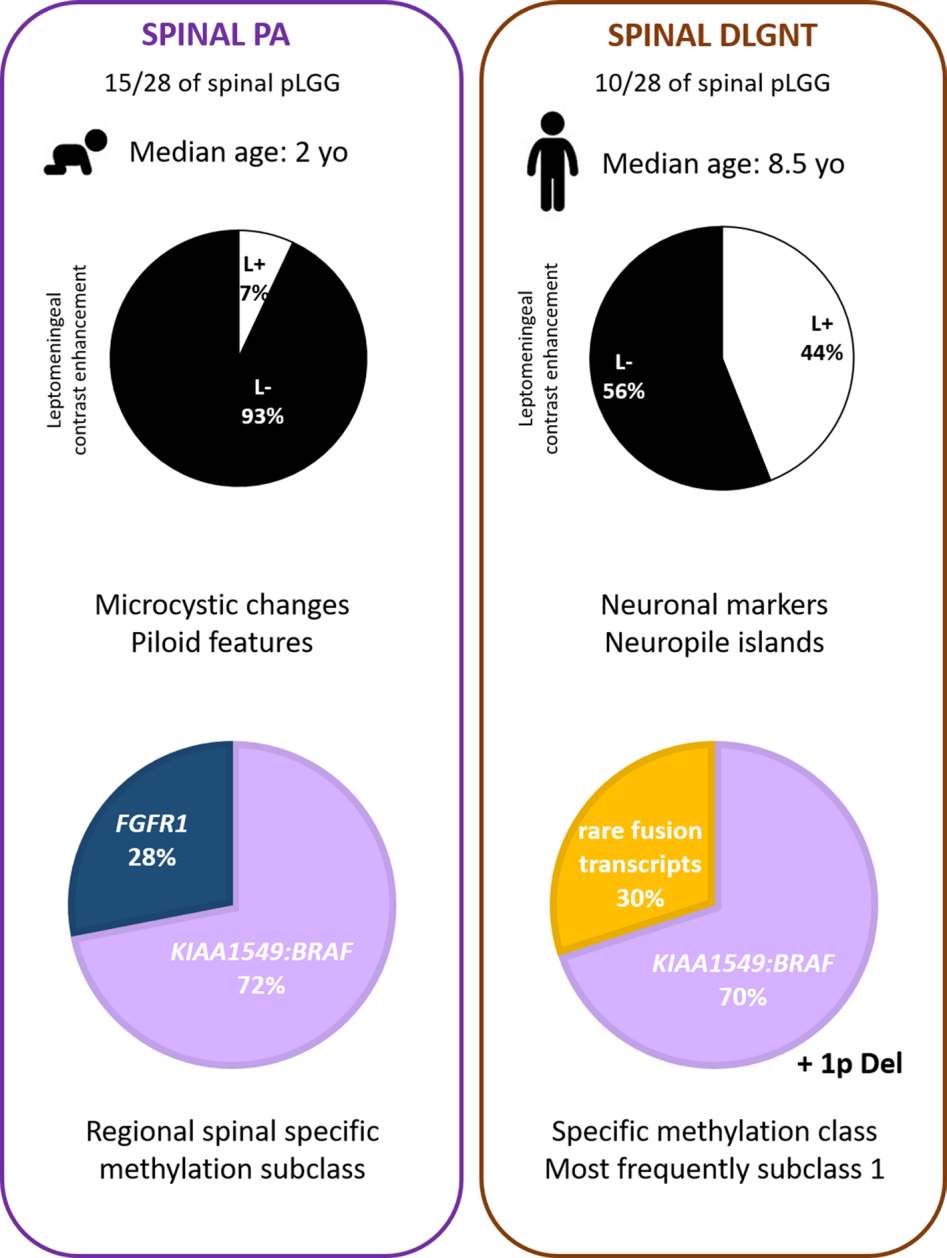
Summary of PA and DLGNT main characteristics. *Del* deletion, *L* leptomeningeal contrast enhancement, *pLGG* pediatric low grade glioma, *yo* years old

DNA methylation profiling classifier from DKFZ subdivides PAs into three subclasses according to their anatomical location: infratentorial, supratentorial and midline, which suggests a regional specificity of DNA methylation profiles in PA [7]. Taken together, our data build on previous works suggesting that PAs from different CNS locations may arise from region-specific progenitors (Fig. 3) [22, 25, 41, 45]. Although MC midline PAs do not include spinal locations, our study is the first to demonstrate that pediatric primary spinal PAs form a unique cluster, distinct from the DKFZ reference cohort, as previously suggested by Lebrun et al. (Figs. 3, 5) [23].

With extensive characterization of spinal tumours including molecular and radiological analysis, our study shows for the first time that a significant proportion of spinal PAs should be reclassified as DLGNTs. We confirmed that 1p deletion is specific to DLGNTs and never occurred in spinal PAs. This was reinforced by one case in this present study with 1p deletion classified as a PA_INF (calibrated score of 0.38), whereas t-SNE reclassified this case as a DLGNT (Figs. 1 and 2, Supplementary Table 2, online resource). When samples are limited, FISH analysis may be useful in assessing 1p status. All DLGNT cases in this series were classified by the v12.5 of the DKFZ classifier as DLGNT subtype 1 which is enriched in pediatric patients and spinal location [12] (Fig. 1 and Supplementary Table 2, online resource). In the present study, *KIAA1549*:*BRAF* is present in spinal PAs and DLGNTs*. RAF1* and *NTRK2* fusions were seen in spinal DLGNTs, as previously described (Fig. 5) [12]. Interestingly, in other studies, these fusions were also documented in PA of other locations than the spine [47, 49]. *FGFR1* TKD alterations were found only in pediatric spinal PAs and not in DLGNTs, suggesting it is an important differential diagnostic criterion [4, 11, 19]. In the literature, *FGFR1* mutation has been reported in only one tumour diagnosed as DLGNT, but this diagnosis was doubtful because of the presence of a H3K27M mutation, the lack of 1p status information, and the absence of DNA methylation profiling [14]. All these results confirm that molecular data (particularly 1p and *FGFR1* status) are crucial in the diagnosis of spinal tumours, whereas histopathology and radiology are of little help (Fig. 5). This study found that the only significant histological variables were microcystic changes, piloid features, and the expression of neuronal markers, which is in line with previous data [10, 48]. Interestingly, neuropil islands were observed in one PA (Fig. 2d, e).

From a clinical perspective, we showed that spinal PAs affect significantly younger patients (median age 2 years old) than DLGNT (median age 8.5 years old) (Table 1 and Fig. 5). In previous published works [4, 6, 8, 23, 36, 40] where the two groups were not differentiated, the median age for spinal LGG ranged from 6 to 14 years. With the exception of age at the time of diagnosis, spinal PAs and DLGNTs show no clear demographic or clinical differences.

Similar to past publications [1, 5], we confirmed in our study that spinal PAs have frequent leptomeningeal involvement (thin leptomeningeal enhancement or nodular metastases) at initial diagnosis (7% of PAs vs 44% of DLGNTs) (Figs. 2, 5). According to the literature, up to 37% of spinal DLGNTs present with a leptomeningeal involvement [12, 17, 21, 26, 27, 35, 38, 39, 44], whereas in the present study, 83% (*n* = 5/6) of epigenetically defined DLGNTs presented initially without leptomeningeal contrast enhancement. This was in line with previous studies suggesting that leptomeningeal dissemination should not be considered as an essential diagnostic criterion for DLGNTs [3, 10, 12]. During the evolution of the disease, progression in PAs and DLGNTs was mostly local, and new metastasis appeared in only one DLGNT case (case #23). The therapeutic strategies in this cohort for spinal LGG were identical to the ones applied to encephalic LGG. None of the first-line strategies took into account the molecular biology. In this retrospective study including mainly tumours initially diagnosed as PA, we found no difference in the type of treatments used for spinal PAs versus DLGNTs. Overall survival could not be used to decipher whether one tumour type of spinal LGG better responds to the treatment, because only one patient died of the disease (a DLGNT). We used the progression-free survival as severity marker, which was not influenced by the tumour type or molecular alterations. Comparing these results with tumours in other locations would, however, be hazardous since the impact and the type of surgery can be strikingly different according to the location. The limitations of the study are the small number of patient and its retrospective nature, which is due to the rarity of these tumour types and the rarity of spinal tumoural locations, resulting in insufficient power of this series. Also, the study includes only pediatric cases and does not allow comparison with adult cases (even rarer).

In summary, pediatric spinal PAs represent a distinct methylation class from PAs in other locations of PA, suggesting that they arise from distinct region-specific progenitors. Because histopathological and radiological criteria remain too similar and not sufficiently discriminant, lack of 1p deletion is essential before proposing a diagnosis of PA in spinal location. The presence of a FGFR1 TKD alteration also favors the diagnostic of PA. Finally, the terms “diffuse” and “leptomeningeal” of DLGNT seem to be a less and less adapted terminology for this tumour type that could be better characterized by the co-occurrence of MAPK and 1p deletion.

## Supplementary Information

Below is the link to the electronic supplementary material.

Supplementary file1 Supplementary table 1: Bibliography (XLSX 11 KB)

Supplementary file2 Supplementary table 2: Data ADP: alive, with disease progression; AND: alive, no evidence of disease; ASD: alive, stable disease; CT: chemotherapy; DLGNT: diffuse leptomeningeal glioneuronal tumour; DLGNT_1: DLGNT subclass 1; DOC: dead of other cause; DOD; dead of the disease; F: female; GG: ganglioglioma; GNT NOS: glioneuronal tumour not otherwise specified; HGG: high-grade glioma; M: male; MC v12.5: methylation class assigned from DNA methylation-based central nervous system tumour classifier version 12.5; NA: not available; NOS: not otherwise specified; oligo NOS: oligodendroglioma not otherwise specified; oligo. comp. PA: oligodendroglial component from a pilocytic astrocytoma; PA: pilocytic astrocytoma; PA_INF: infratentorial pilocytic astrocytoma; PMA: pilomyxoid astrocytoma; SU: surgery; TKD ITD: tyrosine kinase domain internal tandem duplication; TKD mut: tyrosine kinase domain hotspot mutation; vus: variant of unknown signification (XLSX 19 KB)

Supplementary file3 Supplementary table 3: proportion of cases first treated by chemotherapy (CT), surgery (SU) or both (SU+CT) DLGNT: diffuse leptomeningeal glioneuronal tumour; PA: pilocytic astrocytoma (DOCX 13 KB)

Supplementary file4 Supplementary Figure 1: a: CNV plots from DNA methylation profiling data of a spinal pilocytic astrocytoma showing BRAF rearrangement indicated by a narrow gain of the 7q34 region and no 1p deletion. b: CNV plots from DNA methylation profiling data of a diffuse leptomeningeal glioneuronal tumour showing 1p deletion and by a narrow gain of the 7q34 region indicative of BRAF rearrangement (TIF 528 KB)

Supplementary file5 Supplementary Figure 2: a: Rosenthal fibers observed in a pilocytic astrocytoma (case #16). b: microcystic changes in a pilocytic astrocytoma (case #10). c: oligodendroglial-like component in a diffuse leptomeningeal glioneuronal tumour (case #24). d: neuropil island in a diffuse leptomeningeal glioneuronal tumour (case #26). a, b, c: scale bar = 50µm; d: scale bar = 100 µm (TIF 6280 KB)
