# Supplementary material for: Pediatric spinal pilocytic astrocytomas form a distinct epigenetic subclass from pilocytic astrocytomas of other locations and diffuse leptomeningeal glioneuronal tumours
Source: Acta Neuropathol. 2022 Oct 20;145(1):83–95. doi: 10.1007/s00401-022-02512-6 (PMC9582396; doi:10.1007/s00401-022-02512-6)
Supplement: Supplementary file 3 — Supplementary file3 Supplementary table 3: proportion of cases first treated by chemotherapy (CT), surgery (SU) or both (SU+CT) DLGNT: diffuse leptomeningeal glioneuronal tumour; PA: pilocytic astrocytoma (DOCX 13 KB) [file 401_2022_2512_MOESM3_ESM.docx]

Supplementary Table 3: proportion of cases first treated by chemotherapy (CT), surgery (SU) or both (SU+CT)

|  |  | **CT** | **SU** | **SU + CT** | **Total** | **Test** |
| --- | --- | --- | --- | --- | --- | --- |
| *Type* | *DLGNT* | *1 (11.1%)* | *7 (77.8%)* | *1 (11.1%)* | *9 (100%)* | *Test Fisher: p=0.58* |
|  | *PA* | *3 (21.4%)* | *8 (57.1%)* | *3 (21.4%)* | *14 (100%)* |  |

*Note : Information regarding the extent of surgical resection was available in 25 patients. Only 16% (4/25) patients underwent gross total resection whereas 84% (21/25) had only biopsy or partial resection.*
