# Supplementary figures and images for: Pediatric spinal pilocytic astrocytomas form a distinct epigenetic subclass from pilocytic astrocytomas of other locations and diffuse leptomeningeal glioneuronal tumours
Source: Acta Neuropathol. 2022 Oct 20;145(1):83–95. doi: 10.1007/s00401-022-02512-6 (PMC9582396; doi:10.1007/s00401-022-02512-6)

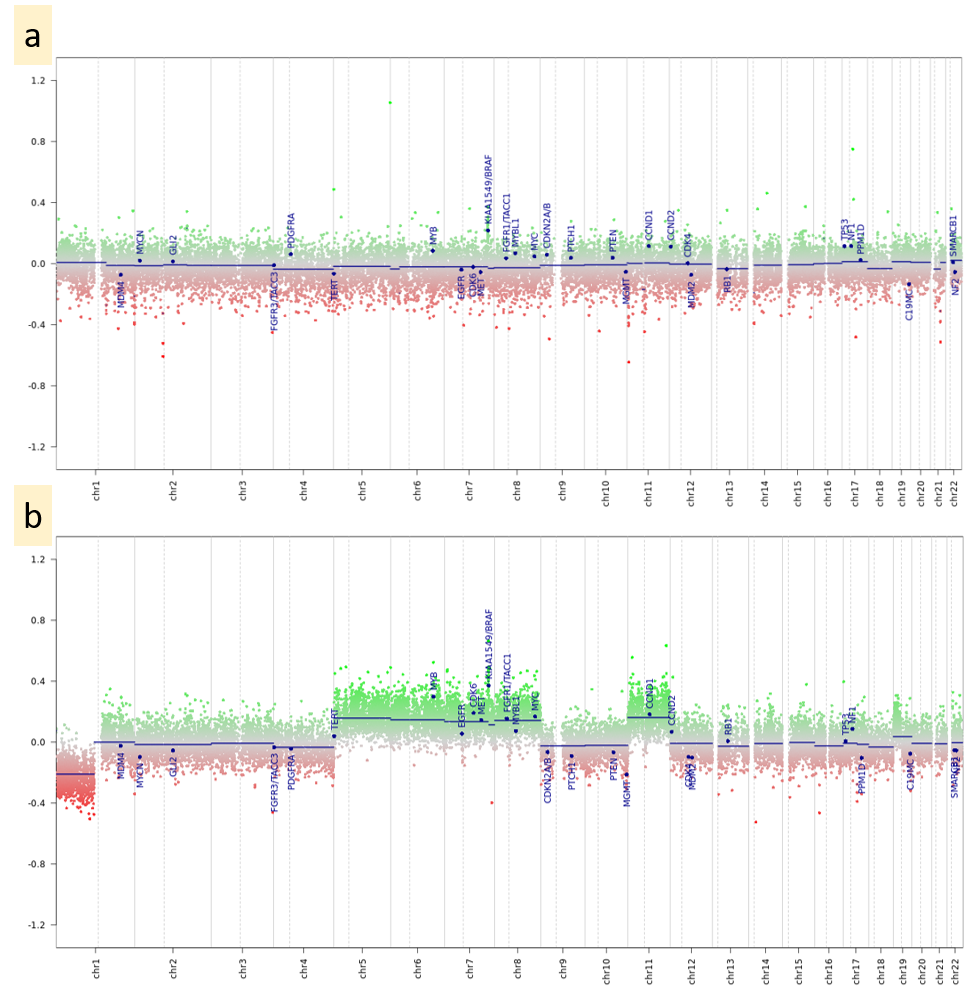

Supplement: Supplementary file 4 — Supplementary file4 Supplementary Figure 1: a: CNV plots from DNA methylation profiling data of a spinal pilocytic astrocytoma showing BRAF rearrangement indicated by a narrow gain of the 7q34 region and no 1p deletion. b: CNV plots from DNA methylation profiling data of a diffuse leptomeningeal glioneuronal tumour showing 1p deletion and by a narrow gain of the 7q34 region indicative of BRAF rearrangement (TIF 528 KB) [file 401_2022_2512_MOESM4_ESM.tif]

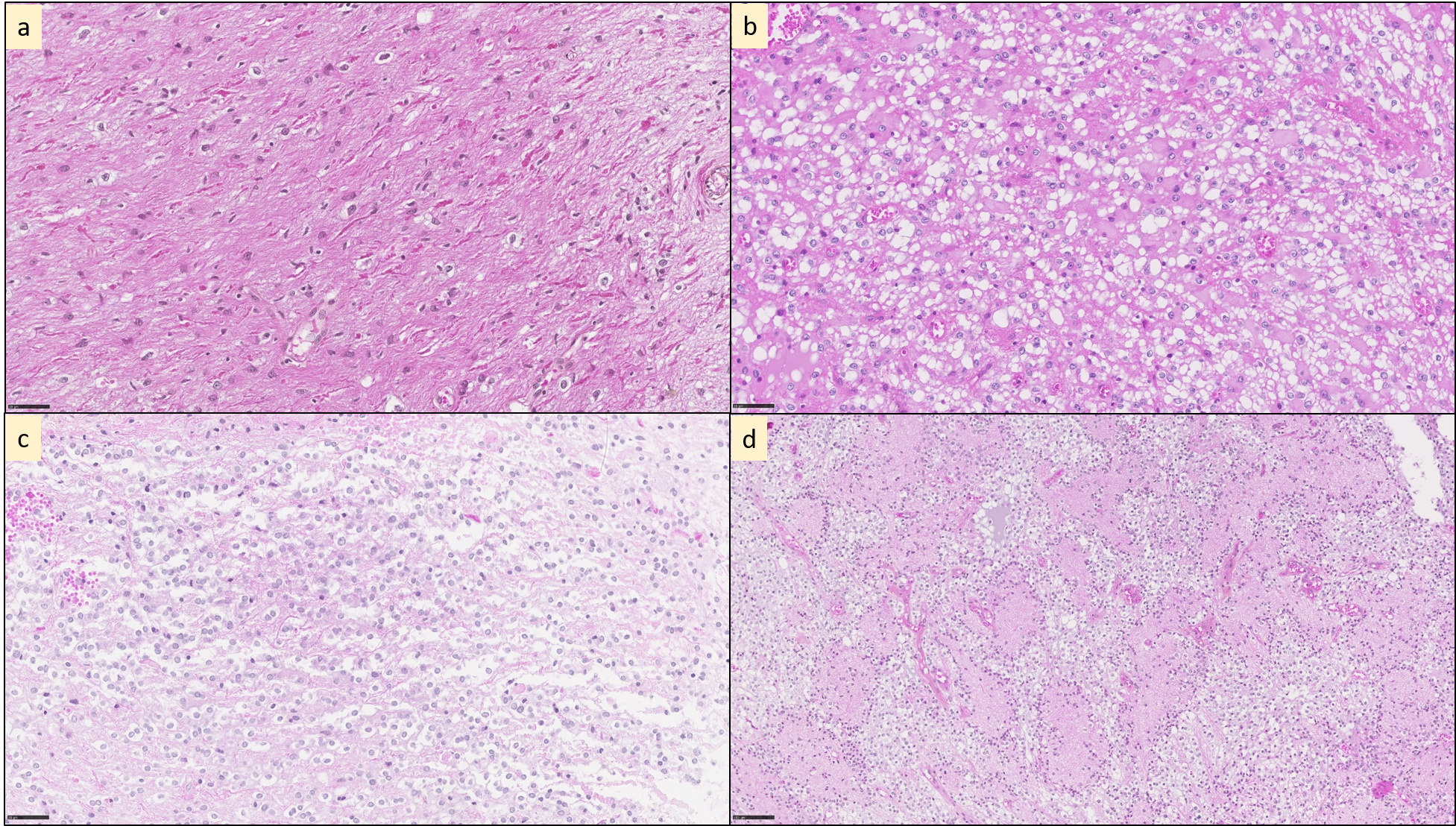

Supplement: Supplementary file 5 — Supplementary file5 Supplementary Figure 2: a: Rosenthal fibers observed in a pilocytic astrocytoma (case #16). b: microcystic changes in a pilocytic astrocytoma (case #10). c: oligodendroglial-like component in a diffuse leptomeningeal glioneuronal tumour (case #24). d: neuropil island in a diffuse leptomeningeal glioneuronal tumour (case #26). a, b, c: scale bar = 50µm; d: scale bar = 100 µm (TIF 6280 KB) [file 401_2022_2512_MOESM5_ESM.tif]
